# Genetics and shell morphometrics of assimineids (Mollusca, Caenogastropoda, Truncatelloidea) in the St Lucia Estuary, South Africa

**DOI:** 10.3897/zookeys.419.7556

**Published:** 2014-06-23

**Authors:** Nelson A. F. Miranda, Ryan van Rooyen, Angus MacDonald, Winston Ponder, Renzo Perissinotto

**Affiliations:** 1 DST/NRF Research Chair in Shallow Water Ecosystems, c/o Department of Zoology, Nelson Mandela Metropolitan University, P O Box 77000, Port Elizabeth, 6031, South Africa; 2 School of Life Sciences, University of KwaZulu-Natal, Westville, Private Bag X5401, Durban, 4000, South Africa; 3 Malacology Section, Australian Museum, 6 College Street, Sydney, 2010, NSW, Australia

**Keywords:** Microgastropods, *Assiminea*, taxonomy, St Lucia Estuary, iSimangaliso Wetland Park

## Abstract

The Assimineidae are a family of amphibious microgastropods that can be mostly found in estuaries and mangroves in South Africa. These snails often occur in great numbers and are ecologically important to the St Lucia Estuary, which forms a crucial part of the iSimangaliso Wetland Park, a UNESCO World Heritage Site. Genetic and shell morphometric analyses were conducted on individuals collected from nine localities distributed from the northern lake regions to the southern lake and the mouth of the St Lucia estuarine lake. Mitochondrial (COI) and nuclear (28S) DNA was used to construct Bayesian Inference, Neighbour-joining, Maximum Parsimony and Maximum Likelihood trees. Principal Component Analysis and Cluster Analysis were performed on standard shell parameter data. Results indicate that two different taxa are present in St Lucia. The taxon comprising individuals from the South Lake and St Lucia Estuary Mouth is identified as *Assiminea* cf. *capensis* Bartsch, in accordance with the latest taxonomic consensus. The taxon comprising assimineid individuals from False Bay, North Lake and South Lake, is here tentatively named “*Assiminea*” aff. *capensis* (Sowerby). These two taxa exhibit patterns of spatial overlap that appear to vary depending on environmental parameters, particularly salinity. The need to resolve the complex taxonomy of assimineids is highlighted.

## Introduction

Assimineidae is a poorly understood family of small amphibious gastropods, belonging to the superfamily Truncatelloidea (previously Rissooidea) (Criscione and Ponder 2013) and are found in estuaries, mangroves, freshwater springs, rivers, streams and terrestrial habitats such as forests, limestone areas and mountain sides (Abbott 1958; Millard and Broekhuysen 1970; Appleton 2002; Fukuda and Ponder 2003; Strong et al. 2008). Some assimineids are recorded inhabiting environments with salinities ranging from freshwater to seawater (Appleton 2002), with a species identified as *Assiminea bifasciata* having been recorded in salinities ranging from 8.3 to 37.6 (Millard and Broekhuysen 1970). Assimineids can be traced to the mid-Tertiary of the Caenozoic Era (65-5 mya, Blair et al. 2001) but probably have a much longer fossil record. They are widely distributed throughout tropical and temperate regions of the world (Abbott 1958). Sometimes referred to as “sentinel snails”, they tend to have brown shells with well-defined spires and are less than 10 mm in height (Appleton 2002). Most assimineids are gonochoristic and it is difficult to distinguish between different species (Appleton 2002). The taxonomy of assimineids is in a constant state of flux at both the specific and generic level. For example, the generic name *Assiminea* is used both broadly and in a more restricted sense (e.g. Fukuda and Ponder 2005, 2006; Hershler et al. 2007). While efforts have been made to genetically and morphologically describe assimineids occurring in some areas of the world (e.g. Abbott 1958; Fukuda and Ponder 2003, 2005), species occurring in South Africa require clarification although partial revisions have been attempted by Connolly (1939), Barnard (1963) and Brown (1994).

The St Lucia Estuary is the largest estuarine system in Africa, a Ramsar site of International Importance and forms part of South Africa’s first UNESCO World Heritage Site, the iSimangaliso Wetland Park (Fielding et al. 1991; Cyrus and Vivier 2006). Lake St Lucia is a rich and diverse biological ecosystem (Pillay and Perissinotto 2008) with a number of endemic species (e.g. Carrasco and Perissinotto 2012; Daly et al. 2012). Assimineids are vitally important to the ecology of the St Lucia Estuary. They are historically a dominant component of the benthic invertebrate assemblages within the system and contribute towards the diet of various higher trophic organisms. Day et al. (1954) lists *Assiminea* sp. as common throughout the estuarine system. Millard and Broekhuysen (1970) reported *Assiminea bifasciata* as common to all areas of the lake, dominating on mud banks, mangroves and aquatic vegetation. This occurred during a period of low salinity. In a survey of the benthic fauna of the St Lucia system following a high salinity period, Boltt (1975) stated that *Assiminea bifasciata* was by far the most dominant species present (in terms of biomass and numbers) and also served as an important dietary component for the macrobenthic community. In a review of benthic surveys in St Lucia, Owen et al. (2010) list *Assiminea* sp. as having been present in all sampling endeavours since 1948. Gut content analyses of iliophagus fish in the St Lucia Estuary revealed that *Assiminea bifasciata* was a significant component of the diet of several species, including *Chanos chanos*, *Mugil cephalus*, *Liza macrolepis* and *Liza dumerilii* (Whitfield and Blaber 1978). *Assiminea* is also one of the main sources of food for the newly described species of sea anemone, *Edwardsia isimangaliso*, which is regarded as micro-endemic to the system (Daly et al. 2012).

While the ecological importance of *Assiminea* is widely recognised, there are inconsistencies in the literature in terms of what species are present in the St Lucia Estuary. Much of the earlier literature refers to *Assiminea bifasciata* as the only species of *Assiminea* present in the system (Day et al. 1954; Millard and Broekhuysen 1970; Boltt 1975; Whitfield and Blaber 1978). *Assiminea sinesis* was also recorded from KwaZulu-Natal in early surveys (Abbott 1958), however subsequent literature makes little reference to this species. More contemporary literature makes mention of *Assiminea ovata* (Miranda et al. 2011; Carrasco et al. 2012; Daly et al. 2012). This is due to a change in the taxonomic status of *Assiminea bifasciata* to *Assiminea ovata*, as reported in Appleton (2002). However, *Assiminea globifera* (Taylor et al. 2006) and *Assiminea durbanensis* (Weerts 1993) have also been reported from St Lucia. One of the latest surveys lists three species of assimineids in St Lucia (MacKay et al. 2010). Other recent studies make reference to *Assiminea* sp. (Pillay and Perissinotto 2008; Owen et al. 2010), supposedly due to the difficulties encountered with identifying the species.

The present study is the first to address the genetics and shell morphometrics of assimineids in the St Lucia Estuary. The aim is to determine the number of distinct taxonomic groups that are present and resolve taxonomic inconsistencies in the literature. This is done by comparing the nuclear and mitochondrial DNA, as well as shell dimensions of individuals of nine different microgastropod populations present inside the system. The specific techniques chosen for this investigation have been used successfully to resolve similar inconsistencies involving assimineids in the Rio Grande region of Mexico (Hershler et al. 2007). It is hypothesised that the assimineids of St Lucia comprise several distinct phylogenetic groups.

## Methods

**Figure 1.**
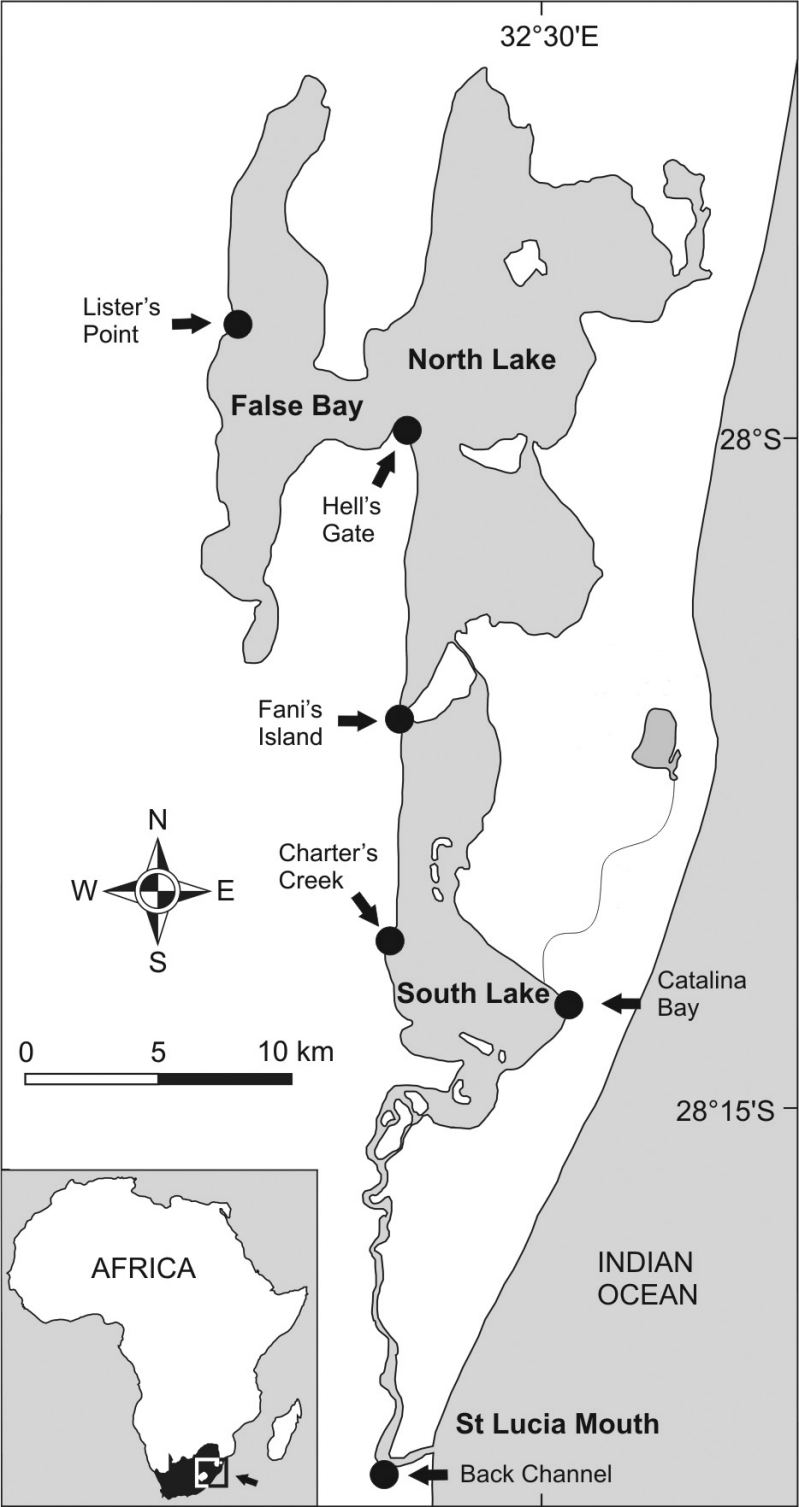
Map of St Lucia Estuary. Sample localities are indicated by arrows and dots (Modified from Miranda et al. 2010).

**Study Site.** The St Lucia Estuary (28°23'S, 32°24'E) covers an area of approximately 350 km^2^ (Taylor et al. 2006) and is Africa's largest estuarine lake (Cyrus 1988). The system is comprised of three large shallow (average 0.9 m depth) lakes, namely False Bay, North Lake and South Lake (Fig. 1). Salinities can range from oligohaline (salinity 0-5) to hypersaline levels (salinity >100) over the course of a number of years (Day et al. 1954; Cyrus 1988, 2010). During periods of low freshwater input and high evaporative water loss, the system exhibits a reverse salinity gradient: a lower salinity can be recorded in the southern regions closer to the mouth, whereas the northern regions of the system tend to become hypersaline. Droughts occur on an almost decadal basis (Taylor et al. 2006). At the time of the present study, St Lucia was emerging from the most severe drought event on record. This dry period ended with freshwater input from Cyclone Irina in March 2012, which led to a system-wide drop in salinity resulting in near marine levels in the northern regions and fresh and brackish conditions prevailing in the southern regions of the system.

**Sampling.** Specimens from 8 populations were collected by net and by hand at 6 localities in the St Lucia Estuary in July and October 2012 (Fig. 1). Snails were collected from two sites at Lister’s Point, False Bay, (Site A: 27°58'10.66"S, 32°23'5.57"E and Site B: 27°58'22.30"S, 32°22'28.24"E). Hell’s Gate (28°0'51"S, 32°26'48"E) and Fani’s Island populations (28°6'34"S, 32°26'03"E) represent the North Lake samples.The False Bay and North Lake region is characterised by highly fluctuating salinities and periodic hypersaline conditions (see Table 1). Charter’s Creek (28°11'52"S, 32°25'05"E) and Catalina Bay (28°13'29"S, 32°29'12"E) samples comprised populations of both larger *Assiminea* cf. *capensis* Bartsch and a smaller species similar to a species originally named *Rissoa capensis* Sowerby and here tentatively named “*Assiminea*” aff. *capensis* (Sowerby) that could thus be separated based on shell height (SH) (see also Table 3). These make up the samples from South Lake, a more stable environment with lower salinities (Table 1) due to higher freshwater input. At the St Lucia mouth, snails were collected from the Back Channel (28°23'45"S, 32°25'09"E) only in October 2012. All specimens were preserved in absolute ethanol prior to analyses.

**Table 1.** Ranges of physico-chemical parameters in the St Lucia Estuary, measured during 2012.

	False Bay and North Lake region	South Lake Western Shores	South Lake Eastern Shores	St Lucia Mouth region
**Temperature (°C)**	19.79–37.49	21.26–34.98	17.56–32.41	15.44–27.29
**Salinity**	39.62–92.20	4.74–12.46	3.07–9.66	8.87–14.42
**pH**	8.27–8.76	8.53–9.14	8.23–9.27	8.30–9.20
**Dissolved oxygen (mg/L)**	5.27–7.96	7.33–9.05	6.18–9.32	6.63–9.23
**Turbidity (NTU)**	144.60–270.45	129.65–308.60	0.70–124.35	16.45–111.83

**Molecular analysis.** Between 4 and 11 individuals from each population were sampled for nuclear and mitochondrial DNA analysis. Genomic DNA was isolated using a Zymogen© extraction kit using the solid tissue protocol and extracted from the remainder of the samples using the entire specimen and following a phenol extraction protocol. Universal primers COIL1490 and COIH2198 were used to amplify and sequence 506 base pair regions of mitochondrial cytochrome c oxidase subunit I (COI) (Folmer et al. 1994). COI amplification followed the protocol: 95 °C for three minutes, followed by 36 cycles (30 seconds at 94 °C, 30 seconds at 40 °C and one minute at 72 °C), followed by a final extension step at 72 °C for 10 minutes. A 728 base pair region of 28S rRNA was amplified and sequenced using D6R and D23F primers (Park and Ó Foighil 2000). The 28S rRNA amplification followed the following protocol: initial denaturing at 95 °C for three minutes, followed by 36 cycles (30 seconds at 94 °C, 30 seconds at 60 °C and one minute at 72 °C) and additional extension at 72 °C for 10 minutes. PCR reactions consisted of 12.5 µl EconoTaq ®, 9.82 µl PCR water, 2 µl Buffer, 1.8 µl MgCL, 1 µl BSA, 0.84 µl of each primer (relevant to the DNA being amplified), 0.2 µl SuperTherm Taq® and 1 µl DNA extract. Some samples required reamplification and 28S rRNA samples required gel extraction.

Sequencing was done at Inqaba Biotech Industries (Pretoria, South Africa) with an ABI 3730 Capillary Sequencer using Big Dye technology. Sequences were edited using BioEdit (v7.0.9.0) (Hall 1999) and haplotypes generated, and molecular diversity indices calculated, with DnaSP (v4.90.1) (Rozas et al. 2003). An AMOVA was performed with GenAlEx (v6.4) in order to determine molecular variance between samples (Peakall and Smouse 2005). Maximum parsimony (MP), neighbour-joining (NJ), maximum likelihood (ML) and Bayesian Inference methods were used to infer phylogenetic relationships. PAUP (v4.0b10) was used to perform the MP, NJ and ML analyses (Swofford 1998) while the Bayesian analyses were done using MrBayes (v3.1.2) (Huelsenbeck and Ronquist 2001). Modeltest (v3.7), under the Akaike Information Criterion (Posada and Crandall 2005), selected a HKY model for construction of the NJ and ML trees (A = 0.275; C = 0.178; G = 0.161; T = 0.387; Ti/tv ratio = 12.438 and rates = equal) for COI and K80 for 28S (A = 0.202; C = 0.257; G = 0.261; T = 0.280; Ti/tv ratio = 2.283 and rates = equal). 1000 bootstrap replicates were used to evaluate node support for trees. The tree bisection-reconnection algorithm was used to generate ML and MP trees. COI trees were rooted with the out-group *Pseudomphala latericea*. 28S trees were rooted with *Pseudomphala miyazakii*, with *Paludinellassiminea japonica* as an additional out-group. Bayesian analyses comprised of two independent runs of four simultaneous Markov Chain Monte Carlo chains. Bayesian analyses were run for 20 000 000 generations and a sample frequency of 1000 generations with a *burn-in* of 25%. The “sump” command was used in MrBayes and Tracer v1.6 (Rambaut and Drummond: http://evolve.zoo.ox.ac.uk/software.html?id=tracer) were used to evaluate the convergence and *burn-in* for likelihood values for post-analysis trees and parameters. The “sumt” command in MrBayes was used to calculate posterior probabilities for trees remaining after *burn-in*.

**Morphometric analysis.** Standard shell parameters were measured and compared between individuals from different localities. Adult individuals were selected from amongst the largest specimen in each sample. Sexual dimorphism was not addressed. 25 individuals from each population were used for morphometrical analysis. Micrographs were taken using a Nikon AZ100 stereo microscope. Snail shells were mounted and orientated with the spiral facing upwards and the aperture facing the optical lens. NIS-Elements (v3.2.00) digital measuring software was used to take measurements. The height and width of the shell (SH, SW), body whorl height (BWH) and aperture height and width (AH, AW) were measured. The number of shell whorls (Whorl) was counted. In addition the ratios of SW/SH, BWH/SH and AH/SH were calculated (Hershler et al. 2007). Primer 6© (v6.1.6) was used to conduct a Principal Component Analysis (PCA) on the normalised data. A SIMPROF cluster analysis using Euclidian distances was carried out in order to group similar samples (Clarke and Gorley 2001).

## Results

**Molecular analysis.** The results of the AMOVA and molecular diversity data are summarised in Table 2. This revealed that for both COI and 28S, the vast majority of the molecular variation (78%) occurred between the populations, with just over 20% occurring within. A high diversity was also found amongst haplotypes but with little variation within. This can be seen by the high haplotype diversity and relatively low variance for both genes (Table 2).

**Table 2.** Molecular diversity and AMOVA data for 28S and COI (*denotes statistical significance).

	28S	COI
**Molecular Diversity Data**		
Haplotypes Generated	18	11
G+C Content	0.633	0.370
Variable Sites	30	16
Parsimony Informative Sites	18	14
Haplotype Diversity	0.769	0.882
Haplotype Variance	0.00305	0.00414
Nucleotide Diversity	0.00788	0.09162
Nucleotide Variance	1.2×10^-6^	6.92×10^-5^
Tajima's D	-0.83178	2.497
**AMOVA**		
Variance among populations	78%*	96%
Variance within populations	22%*	4%

The COI data set yielded 11 haplotypes (Table 2) with 98 variable sites, 87 of which were parsimony informative. The average G+C content was 37%. Haplotype 1 was the most common and was represented by individuals from both Lister’s Point sites. All other haplotypes were represented by 1-3 individuals from single populations.

**Figure 2.**
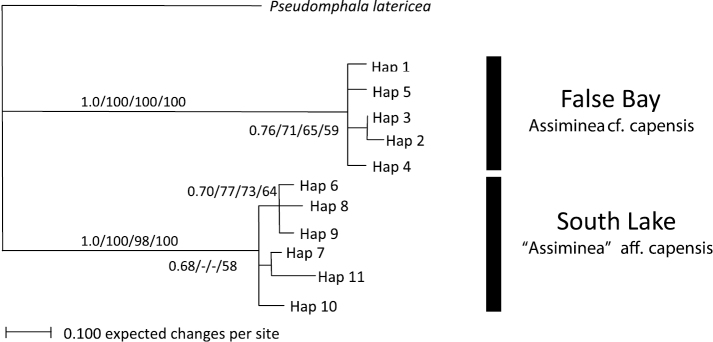
Bayesian Inference tree for COI data. Support values are as follows: Bayesian Inference/NJ/ML/MP.

All phylogenetic analyses strongly supported the separation of the False Bay (Lister’s Point, i.e. “*Assiminea*” aff. *capensis* (Sowerby)) and South Lake (Charter’s Creek and Catalina Bay, i.e. *Assiminea* cf. *capensis* Bartsch) samples into distinct clades, with supporting bootstrap values of 100% NJ and MP, 98% for ML and 1.0 for Bayesian inference (Fig. 2). All trees showed weak to moderate support for sub clades including Haplotype 2 and 3. All trees showed weak to moderate support for a Haplotype 6, 8 and 9 sub clade and Bayesian inference and MP trees for a Haplotype III and VI sub clade. The average variance between the two regions was 86.23 base pairs, which equates to a 17.04% genetic variance (Table 2).

**Figure 3.**
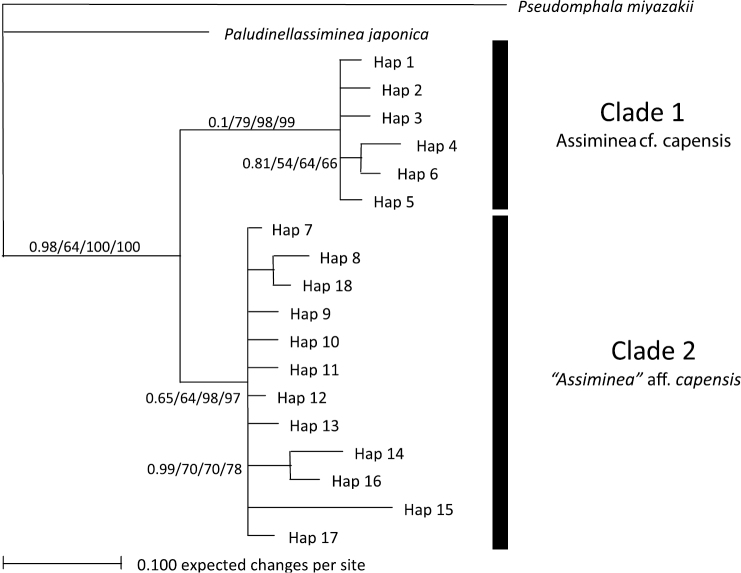
Bayesian Inference tree for 28S data. Support values are as follows: Bayesian Inference/ML/MP/NJ.

The 28S data set yielded 18 haplotypes (Table 2) with 30 variable sites, 18 of which were parsimony informative. The average G+C content was 63.3%. Haplotype 7 was the most common (27 individuals) and was represented by all individuals from all populations, except the large Charter’s Creek, large Catalina Bay and Back Channel individuals. Phylogenetic analyses showed varying support for the separation of two clades (Fig. 3). The first clade was made up entirely of all the individuals from the large Charter’s Creek, large Catalina Bay and Back Channel individuals (i.e. *Assiminea* cf. *capensis* Bartsch). This clade was strongly supported by all phylogenetic analyses with the exception of ML. The MP and NJ trees showed strong support for the separation of a second clade. This clade was made up entirely of all samples from the other four populations (i.e. “*Assiminea*” aff. *capensis* (Sowerby)). All trees differed only slightly in the position of the branches within the clades.

**Figure 4.**
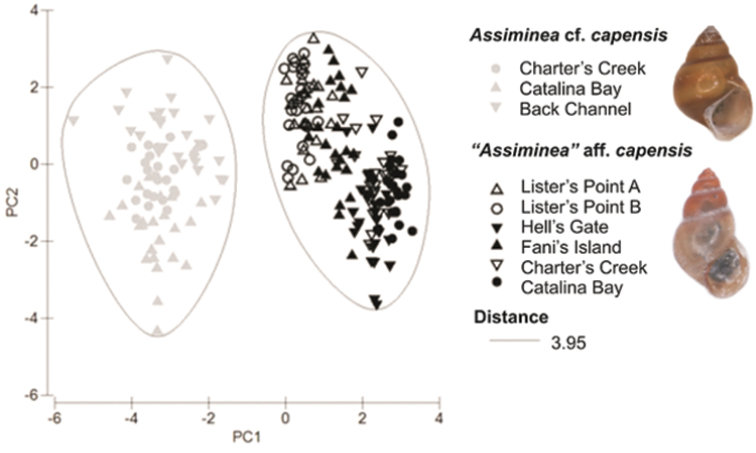
Plot of first two principal components of PCA for morphometric data. The cluster analysis ellipses are associated with *P* = 0.001.

**Morphometric analysis.** Generally larger shell sizes (SH, SW, BWH, AH and AW) were recorded for *Assiminea* cf. *capensis* Bartsch in comparison to “*Assiminea*” aff. *capensis* (Sowerby) (Table 3). Adult *Assiminea* cf. *capensis* also tended to have a greater number of whorls (Whorl) (Table 3). However, the *Assiminea* cf. *capensis* populations from Back Channel, Charter’s Creek and Catalina Bay were grouped separately from the “*Assiminea*” aff. *capensis* populations at Lister’s Point, Hell’s Gate, Fani’s Island, Charter’s Creek and Catalina Bay, on the basis of shell morphometry in the cluster analysis (Fig. 4). The first two principal components (PCs) accounted for 89.1% of the total variation in shell morphometry (Table 4). All the shell dimensions recorded loaded high in PC1 and the ratios loaded high in PC2. This would suggest that samples are separated primarily on the basis of size (PC1) and shape (PC2).

**Table 3.** Shell parameters (mean ± standard deviation) of populations of *Assiminea* cf. *capensis* and “*Assiminea*” aff. *capensis* occurring in the St Lucia Estuary during 2012.

Parameter	*Assiminea* cf. *capensis*			“*Assiminea*” aff. *capensis*				
	Charter’s Creek	Catalina Bay	Back Channel	Lister’s Point	Hell’s Gate	Fani’s Island	Charter’s Creek	Catalina Bay
SH	3.82 ± 0.25	3.99 ± 0.32	3.86 ± 0.66	2.22 ± 0.18	1.22 ± 0.19	1.70 ± 0.20	1.26 ± 0.12	1.07 ± 0.06
SW	2.52 ± 0.16	2.73 ± 0.19	2.42 ± 0.35	1.30 ± 0.89	0.81 ± 0.10	1.04 ± 0.11	0.79 ± 0.06	0.67 ± 0.03
BWH	2.67 ± 0.16	2.90 ± 0.24	2.70 ± 0.43	1.52 ± 0.12	0.92 ± 0.13	1.22 ± 0.13	0.93 ± 0.07	0.83 ± 0.05
AH	1.81 ± 0.15	1.97 ± 0.17	1.85 ± 0.26	0.98 ± 0.08	0.61 ± 0.08	0.75 ± 0.08	0.58 ± 0.06	0.49 ± 0.03
AW	1.59 ± 0.14	1.73 ± 0.15	1.47 ± 0.21	0.81 ± 0.06	0.49 ± 0.07	0.63 ± 0.06	0.48 ± 0.05	0.41 ± 0.02
Whorl	6.00 ± 0.01	4.80 ± 0.41	6.04 ± 0.20	4.94 ± 0.33	4.00 ± 0.01	4.80 ± 0.41	4.20 ± 0.41	3.92 ± 0.28
SW/SH	0.66 ± 0.03	0.68 ± 0.03	0.63 ± 0.03	0.59 ± 0.03	0.67 ± 0.04	0.62 ± 0.04	0.63 ± 0.02	0.63 ± 0.03
BWH/SH	0.70 ± 0.02	0.73 ± 0.03	0.70 ± 0.02	0.69 ± 0.03	0.76 ± 0.04	0.72 ± 0.04	0.75 ± 0.03	0.77 ± 0.03
AH/SH	0.42 ± 0.02	0.433 ± 0.02	0.38 ± 0.02	0.37 ± 0.02	0.40 ± 0.03	0.37 ± 0.03	0.38 ± 0.03	0.39 ± 0.02

Measuring units are in mm. Sample size is 25 for each population and locality.

**Table 4.** Factor loadings from principal component analysis for normalised data.

Variable	PC1	PC2	PC3	PC4	PC5
SH	**-0.407**	0.048	-0.147	0.106	-0.057
SW	**-0.408**	-0.038	-0.097	0.082	-0.134
BWH	**-0.406**	-0.001	-0.231	0.091	-0.044
AH	**-0.407**	-0.021	-0.16	0.074	-0.056
AW	**-0.408**	-0.049	-0.077	0.167	0.028
Whorl	-0.326	0.251	0.184	**-0.823**	**0.345**
SW/SH	-0.102	**-0.602**	**0.328**	**-0.334**	-0.63
BWH/SH	0.179	**-0.495**	**-0.761**	**-0.305**	**0.205**
AH/SH	-0.148	**-0.568**	**0.405**	0.242	**0.644**
Eigenvalues	5.91	2.11	0.441	0.289	0.239
% Cum Var	65.7	89.1	94	97.2	99.9

## Discussion

Molecular (COI and 28S) as well as shell morphometric analyses support a clear distinction of two clades within the assimineids occurring in the St Lucia estuarine lake during the study period (Figures 2–4). There is a large divergence in mitochondrial DNA (17.04%) and the variance in the slower evolving nuclear rDNA between the two clades. The divergence in the COI gene is greater than that previously shown for other congeneric truncatelloidean gastropods, which ranged from 1.1 to 14.8% (e.g. Hershler et al. 1999; Liu and Hershler 2005; Hershler et al. 2006, 2007). At least two distinct species exist in the St Lucia Estuary. These two separate species have tentatively been identified as *Assiminea* cf. *capensis* Bartsch (the larger-sized Charter’s Creek, Catalina Bay and Back Channel populations) and “*Assiminea*” aff. *capensis* (Sowerby) (the smaller-sized Lister’s Point, Hell’s Gate, Fani’s Island, Charter’s Creek and Catalina Bay populations) (W P pers. obs.). Although on average *Assiminea* cf. *capensis* is larger than “*Assiminea*” aff. *capensis*, both species have variable shell morphology and multivariate analyses are required to make clear distinctions based on morphometry alone (Fig. 4, Table 3). Shell size ratios (SW/SH, BWH/SH and AH/SH) are not useful to differentiate between the species (Table 3). *Assiminea* cf. *capensis* may be the taxon previously recognised as *Assiminea ovata* and *Assiminea bifasciata* from the St Lucia Estuary and possibly generally in South Africa (e.g. Kilburn and Rippey 1982).

Environmental conditions, salinity in particular, strongly influence the spatial and temporal distribution and overlap patterns of *Assiminea* cf. *capensis* and “*Assiminea*” aff. *capensis* in the St Lucia Estuary. In an earlier benthic survey of St Lucia conducted by Boltt (1975), an initial sampling effort was conducted under high salinity conditions and *Assiminea bifasciata* (= *Assiminea* cf. *capensis* Bartsch) was not found in False Bay. Following an extended period of freshwater input, salinity dropped from levels as high as 80 to below that of sea water. Subsequent sampling efforts demonstrated the rapid recolonisation of False Bay and North Lake by *Assiminea bifasciata* (Boltt 1975). According to the present study, *Assiminea* cf. *capensis* was not found in the highly saline False Bay area between November 2011 and February 2012 (salinity 56–92). In March 2012, high freshwater input associated with Cyclone Irina, caused salinity in False Bay to drop (to about 30) and a rapid population boom of “*Assiminea*” aff. *capensis* was recorded at the same time. Furthermore, in July 2013, both *Assiminea* cf. *capensis* and “*Assiminea*” aff. *capensis* were found at Lister’s Point. Taylor et al. (2006) suggested that *Assiminea* cf. *capensis* take refuge in freshwater seepage areas in the South Lake under drought conditions. By acting as a constant source of freshwater, ground water plays a role in stabilising salinity and water level conditions in the South Lake (Vrdoljak and Hart 2007). It is there that both “*Assiminea*” aff. *capensis* and *Assiminea* cf. *capensis* co-exist in Charter’s Creek and at Catalina Bay. Both species can avoid desiccation and hypersaline conditions by inhabiting freshwater refugia and recolonize the system when optimal environmental conditions are re-established (as reported by Millard and Broekhuysen 1970). However, it is hypothesised that “*Assiminea*” aff. *capensis* has wider environmental tolerance limits (particularly upper salinity limits, allowing it to also persist in False Bay), compared to *Assiminea* cf. *capensis*.

Given the morphological and ecological similarities, as well as spatial overlap displayed by *Assiminea* cf. *capensis* and “*Assiminea*” aff. *capensis*, it is not surprising that both species have been misidentified in the past. Millard and Broekhuysen (1970) reported the presence of *Assiminea bifasciata* in soft mudbanks and mangrove forests at salinities ranging from 8.3 to 37.6. These authors also reported an expansion of its range from South Lake in July 1964 to False Bay by January 1965, coinciding with an overall significant rise in salinity. Interestingly, they also reported the occurrence of another microgastropod, *Syncera* sp., in South Lake and False Bay at salinities ranging from 36.0 to 52.6 (“on *Zostera* and on banks”). *Syncera* Gray, 1821 is *nomen nudum* and treated as a synonym of *Assiminea* (Fukuda and Ponder 2003). Due to coincidences in terms of the general shape of the shell, the locations where specimens were found and their reported salinity range which extends to hypersaline conditions, it is likely that the species that Millard and Broekhuysen (1970) referred to as *Syncera* sp. was actually “*Assiminea*” aff. *capensis*. “*Assiminea*” aff. *capensis* has been referred to as *Coriandria durbanensis* and *Assiminea durbanensis* in previous studies (Weerts 1993; Raw et al. 2013). However, “*Assiminea*” aff. *capensis* is of unknown relationship since the 1980s and *Coriandria durbanensis* was even mistakenly suggested to be a member of the Hydrobiidae (Ponder and Yoo 1980). The specimens tentatively named “*Assiminea*” aff. *capensis* in the current study are assimineids but in an as yet unnamed genus. There is clearly a great need for taxonomic revision based on comparative anatomy and molecular analysis.
